# Cortical hyperarousal during sleep in mice receiving BACE inhibitors may affect cognitive performance

**DOI:** 10.1002/alz.094704

**Published:** 2025-01-09

**Authors:** Elyse A Watkins, Christopher J Olker, Xuanyi Lin, Eun Joo Song, Sarah Kim, Fred W Turek, Matthew E. Kennedy, Martha H Vitaterna, Robert J. Vassar

**Affiliations:** ^1^ Northwestern University, Chicago, IL USA; ^2^ MRL, Merck & Co., Inc., Boston, MA USA; ^3^ Feinberg School of Medicine, Northwestern University, Chicago, IL USA

## Abstract

**Background:**

BACE inhibitors, while effective in lowering amyloid‐beta production, have been associated with mild cognitive worsening in clinical trials. Additional treatment‐related adverse events reported in multiple clinical trials were sleep disturbances and insomnia. The purpose of this study is to determine if sleep disturbances occur in mice receiving BACE inhibitor, if sleep disturbances correlate with cognitive impairment, and the mechanism by which this may occur.

**Method:**

Adult C57BL/6 mice were treated with high dose BACE inhibitor (Merck) or control chow for 12 weeks. At 12 weeks, mice were implanted with a head mount containing electrodes for EEG and EMG monitoring. Sleep was monitored for 48 hours continuously. Cognitive assessments including object location memory, Y‐maze, and Barnes maze were performed. 10 second sleep epochs were manually scored as “wake”, “REM”, and “non‐REM”, based on EEG and EMG, to then train an algorithm to detect and define all remaining epochs. Fast Fourier transforms were performed on all epochs to identify the relative delta, theta, alpha, sigma, beta, and gamma power.

**Result:**

Mice receiving BACE inhibitor chow had altered sleep patterns. This was marked by fewer, longer REM bouts than control mice. Additionally, mice receiving BACE inhibitor had less slow wave non‐REM sleep, marked by a reduction in delta power. Conversely, mice had an increase in the higher frequency beta and gamma waves during non‐REM sleep, suggestive of cortical hyperarousal.

**Conclusion:**

Mice receiving BACE inhibitors for three months had impaired sleep. Longer REM bouts could point to the vivid dreams and disturbed sleep noted in humans. A reduction in the slow wave sleep indicates reduced restorative sleep, which could impair performance on cognitive tests by leading to lethargy and impaired memory consolidation. Furthermore, cortical hyperarousal during non‐REM sleep is prevalent in a number of disorders in humans, including insomnia, anxiety, and post traumatic stress disorder, which could help elucidate an underlying mechanism. This study has implications for the future of clinical trials, because there are commercially available drugs that could restore normal non‐REM sleep and potentially alleviate cognitive worsening.

